# The health of the elderly and social security in the context of digital financial inclusion in China

**DOI:** 10.3389/fpubh.2022.1079436

**Published:** 2023-01-09

**Authors:** Lei Xiao, Yanyan Wu, Xin Cao

**Affiliations:** ^1^School of Economics and Management, Kunming University, Kunming, Yunnan, China; ^2^Pan-Asia Business School, Yunnan Normal University, Kunming, Yunnan, China

**Keywords:** the health of the elderly, DFI, social security, PSM–DID, policy effects

## 1. Introduction

China's economy outperforms those of other nations. However, China's population aging issue is getting progressively worse. Due to its rapidly aging population, China's society is under extreme pressure.

According to China's 14^th^ 5-Year Plan, it should create a multitiered, equitable, and sustainable social security system that protects the entire population in city, urban, and rural areas. The growth of the Internet has significantly altered the Chinese financial sector. Consequently, China is gradually making the transition from traditional financial inclusion to digital financial inclusion (DFI). DFI can greatly raise the participation rate in social security. The question remains, then, what the connection is between the health of the elderly and DFI. This article will conduct a discussion on this subject.

## 2. Literature review

Previous studies generally concentrate on two aspects of how social security affects the health of the elderly.

First, both public pensions and medical insurance may increase the utilization of healthcare services and medical examinations, thus, improving people's health (1–3). Meanwhile, many studies have found that public pensions are positive for the health of the elderly (4–6).

With the growth of DFI in China, its influence cannot be ignored, although few studies have examined how social security affects the health of the elderly in the context of DFI. Therefore, it is important to investigate whether social security has beneficial effects on the health of the elderly in the context of DFI.

Second, concerning the heterogeneity of its impact on the health of the elderly, early research discovered that socioeconomic status inequalities were associated with differences in consumption of health services or health status (7–9). Nevertheless, there is limited evidence on the impact of social security on health among middle-aged and elderly people in rural China. Therefore, social security should be reformed to enhance its positive impact on health (10). Due to China's unique health insurance system, there are significant gender differences in health coverage, especially among the elderly (11). Although women tend to live longer than men, many studies have shown that they are in poorer health (12–14).

Most scholars have studied how the social security system affects the health of the elderly. Polsky et al. discovered that the occurrence of self-rated good health improves after the elderly join a health insurance plan (15). Card et al. found no significant effect of enrollment in an old-age health insurance plan on mortality (16). Higher levels of trust have a positive effect on individuals' health status (17). By improving their income status, the social security system has a positive impact on the health status of the elderly (18, 19).

Previous studies have offered a comprehensive overview and can be described as follows: (1) Most studies focus on how social security affects the health of the elderly, but they rarely examine how DFI affects the health of the elderly. This study uses instrumental activities of daily living (IADL) and activities of daily living (ADL) to measure the factors that affect the health of the elderly with the use of the Chinese Longitudinal Healthy Longevity Survey (CLHLS) database. (2) Concerning regional heterogeneity, this article categorizes the samples into different groups according to the place of residence of the respondents. (3) The PSM–DID model is used to analyze the various effects of social security on the health of the elderly during various eras of DFI.

## 3. Data sources and research methodology

### 3.1. Data sources

First, DFI in China is provided by Peking University Digital Finance Research Center and Ant Financial company. Second, the health level of the elderly is from the four follow-up surveys in CLHLS in 2011 and 2018 conducted by PKU Center for Healthy Aging and Development.

Considering the poor amount of data of those aged above 105 years, data from the elderly aged 65–105 years were selected. After the elimination of missing values and unsuitable answers, 14,887 samples were finally chosen.

### 3.2. Research methodology

Propensity score matching–difference-in-differences, proposed by Heckman et al. (20), is often used to evaluate the impact of policies in recent years as it can reject endogeneity problems caused by unobservable variables through propensity score matching. Thus, this article selected PSM–DID to solve the endogeneity problems caused by sample self-selection and omitted variables while controlling the different impacts of the observable variables on the treatment and control groups to measure the average treatment effect of the elderly who participate in social security.

### 3.3. Model setting

The elderly who participated in social security in 2018 were included in the treatment group. In contrast, those who participated in social security in 2011 but did not do so in 2018 were included in the control group. To guarantee the validity of the empirical findings, missing and erroneous data were removed from the dataset. The fundamental structure of the model is as follows:


(1) $$\begin{array}{l} Health_{i,t}\;=\;\alpha_{0}+\alpha_{1}\;*\;did_{i,t}+\alpha_{2}\;*\;Control_{i,t}+\varepsilon_{i,t} \end{array}$$

where *i* represents the data for the treatment group and the control group, *t* represents different time points and the independent variable *did*_*it*_ is a difference-in-differences variable such that *did*_*it*_ = *treat*_*it*_ * *time*_*it*_. Referring to the principle of setting the difference-in-differences variable, this article selected the elderly who participated in social security in 2018 as the treatment group. If they participated in social security, *treat*_*it*_ = 1; otherwise, *treat*_*it*_ = 0. In addition, this article took the development of DFI in 2018 as the benchmark and assigned *time*_*it*_ as 1 in 2018 and 0 in 2011.

The dependent variable is the health of the elderly, which is measured from the two dimensions of IADL and ADL. IADL is formed by the questions included in the questionnaire. We considered those who answered “Yes” to eight questions as having perfect IADL scores (IADL = 1); otherwise, the IADL score is imperfect (IADL = 0). ADL is formed similarly. We regarded those who answered “Yes” to six questions as having perfect ADL scores (ADL = 1); otherwise, the ADL score is imperfect (ADL = 0). Finally, IADL and ADL were summed to form a single health variable: if both IADL and ADL are assigned as 1, the health variable is assigned as 1; otherwise, the health variable is assigned as 0.

Considering the impact of participating in social security and self-reported health, age, income status, marriage status, access to medical care, and the number of children are regarded as individual and family characteristic variables.

## 4. Regression results

### 4.1. Matching quality test on samples

We used PSM–DID to evaluate the impact of social security on the health of the elderly in the context of DFI.

After calculating the propensity score using the logit model, we checked the sample matching accuracy. We examined two fundamental propensity score hypotheses. The balance hypothesis is one of the two fundamental hypotheses. After matching, practically all control variable biases in the treatment and control groups were decreased by more than 50%, which shows that there is no discernible difference in the observable variables between the treatment and control groups.

### 4.2. Full sample

Supplementary Table 1 shows that social security can significantly improve the health of the elderly at the 1% level in the context of DFI in Model 1 before adding control variables. When other control variables are added (Model 2), social security can still significantly improve the health of the elderly at the 1% level. Moreover, in Model 2, the R-squared increases from 23.3 to 24.6%. After combining Model 1 and Model 2, the *did*_*i,t*_ is significantly positive for the health of the elderly, that is, with the development of DFI, the elderly who participate in social security are healthier than those who do not.

### 4.3. Sex group subsamples

In this section, we categorize the samples into male and female groups and analyze the impact of social security on the health of elderly men and women. The results are shown in Supplementary Table 2.

Supplementary Table 2 reports that social security has different impacts on elderly men and women in terms of the development of DFI. Compared with Model 3 and Model 4, social security is more positive for elderly men (Model 3) than for elderly women (Model 4). Since men are more likely to suffer than women from occupation-related fatty liver disease, strokes, and chronic lung disease, elderly men participate in social security more actively than women. Moreover, the social division of labor caused by traditional gender norms requires men to have better economic status to maintain good health than women, while women's non-market labor is uncompensated and takes a toll on their health.

### 4.4. Residence subsamples

In this section, we analyze the place of residence subsamples, that is, city, urban, and rural. The regression result of the DID model is shown in Supplementary Table 2.

Supplementary Table 2 shows that for these residence subsamples, after adding the control variables, social security has significantly different impacts on the health of the elderly. By comparing all models, it can be seen that social security has a significantly positive impact on the health of the elderly in all places of residence. However, according to the different impacts of social security on the city, urban, or rural elderly (i.e., Model 5, Model 6, and Model 7), in the context of DFI, it has the most positive impact on the rural elderly. DFI will thus help rural areas improve their public services. Improving basic public services such as education, social security, and medical care will greatly narrow the health gap between urban and rural areas and increase farmers' economic wellbeing. DFI is, therefore, an important form of rural public service infrastructure that can provide rural residents with comprehensive financial services.

Since DFI is obtained by weighing three subindices of health coverage, depth of use, and digitization, we incorporate each subindex of DFI in the model to analyze their impact on the health of the elderly to further explore how to integrate DFI into the pension insurance system in rural areas. The breadth of coverage reflects the degree of coverage of digital financial services. The wider the coverage, the more it helps the elderly to obtain financial services. Depth of use reflects the actual application of DFI with deeper use, indicating that seniors have access to high-quality financial services that meet their different financial needs.

Based on the aforementioned discussion, the local government is advised to expand coverage and deepen the use of digital finance to improve DFI in rural areas. If DFI is improved in rural areas, social security will have a more positive impact on the health of the elderly.

## 5. Discussion and conclusions

As mentioned previously, our results demonstrate that enhancing the social security system in the context of DFI is positive for the health of the elderly who participate in it. Social security is much more beneficial for elderly men than women and has the most positive impact on the health of those who live in rural areas.

In the context of the Healthy China strategy, improving the health of the rural elderly has attracted much attention from the government and academia. There are significant differences in how to enhance the health of various elderly groups. The government should combine DFI with social security to improve the health of the elderly. Furthermore, since different areas have varying economic development levels and insurance programs, the government should provide financial assistance to the elderly who are in need of social security.

Moreover, the government is required to improve basic services and medical care to support DFI because it is an important form of rural public service infrastructure that offers rural residents access to a full range of financial services, including payments, credit, insurance, and financial management.

Considering that few studies have studied the impact of DFI on the participation rate of the elderly in social security health of the elderly, the aforementioned findings are conducive to advancing the relevant studies in this area and provide a basis for the formulation and implementation of social security policies in the future.

Most studies focus on how social security affects the health of the elderly, while this article focuses on how DFI affects it and analyzes the gender and regional heterogeneity of the influence of DFI on the health of the elderly in different periods in depth.

The limitations of this study are as follows. First, due to the problem of data acquisition, only two periods of data can be sourced. Second, since the educational level of the respondents cannot be accurately obtained, their educational heterogeneity cannot be studied.

## Data availability statement

Publicly available datasets were analyzed in this study. This data can be found here: https://opendata.pku.edu.cn/dataset.xhtml?persistentId=doi:10.18170/DVN/WBO7LK&version=2.0.

## Author contributions

LX has designed the research and conducted the original analyses. YYW has re-conducted the analyses required by the reviews and made significant contributions to the revision of the paper. XC has participated in the revision of the paper and made significant contributions to the literature review. All authors contributed to the article and approved the submitted version.
