# Supplementary material for: The health of the elderly and social security in the context of digital financial inclusion in China
Source: Front Public Health. 2023 Jan 9;10:1079436. doi: 10.3389/fpubh.2022.1079436 (PMC9868769; doi:10.3389/fpubh.2022.1079436)
Supplement: Supplementary file 1 [file Table_1.DOCX]

Supplementary Material

The health of the elderly and social security in the context of digital financial inclusion in China

Lei Xiao*, Yanyan Wu, Xin Cao

*** Correspondence:** Lei Xiao: leisureostrich@126.com

# Supplementary Tables

**Table 1 |** The regression results of the difference-differences model under the overall samples.

|  | Model 1 | Model 2 |
| --- | --- | --- |
| Variables | health | health |
| did | 0.165*** | 0.154*** |
|  | (16.64) | (18.12) |
| The Control Variables | N | Y |
| Individual fixed effects | Y | Y |
| N | 14,711 | 14,711 |
| R squared | 0.233 | 0.246 |

Robust t-statistics in parentheses, *** p<0.01, ** p<0.05, * p<0.1. The control variables are age, income status, the number of children, marriage status, access to medical care.

**Table 2 |** The regression results of the difference-differences model under the samples of different sex groups and city/urban/rural areas.

|  | male | female | city | urban | rural |
| --- | --- | --- | --- | --- | --- |
|  | Model 3 | Model 4 | Model 5 | Model 6 | Model 7 |
| Variables | health | health | health | health | health |
| did | 0.314*** | 0.202*** | 0.106*** | 0.136*** | 0.178*** |
|  | (10.80) | (13.53) | (5.245) | (9.534) | (14.02) |
| The Control Variables | Y | Y | Y | Y | Y |
| Individual Fixed Effects | Y | Y | Y | Y | Y |
| N | 3,962 | 6,304 | 3,264 | 5,207 | 6,245 |
| R squared | 0.265 | 0.287 | 0.257 | 0.263 | 0.246 |

Robust t-statistics in parentheses, *** p<0.01, ** p<0.05, * p<0.1. The control variables are age, income status, the number of children, marriage status, access to medical care.
